# Drivers of Emerging Infectious Disease Events as a Framework for Digital Detection

**DOI:** 10.3201/eid2108.141156

**Published:** 2015-08

**Authors:** Sarah H. Olson, Corey M. Benedum, Sumiko R. Mekaru, Nicholas D. Preston, Jonna A.K. Mazet, Damien O. Joly, John S. Brownstein

**Affiliations:** Wildlife Conservation Society, New York, New York, USA (S.H. Olson);; University of Wisconsin Madison, Madison, Wisconsin, USA (S.H. Olson);; Boston University School of Public Health, Boston (C.M. Benedum);; Boston Children’s Hospital, Boston, Massachusetts, USA (C.M. Benedum, S.R. Mekaru, N.D. Preston, J.S. Brownstein);; University of California, Davis, California, USA (J.A.K. Mazet);; Metabiota, Nanaimo, British Columbia, Canada (D.O. Joly);; Harvard Medical School, Boston (J.S. Brownstein)

**Keywords:** epidemiology, communicable diseases, emerging, public health, data collection, awareness, disease outbreaks, risk, the Internet, surveillance, detection, digital disease detection, disease drivers, antecedent conditions, disease events, epidemic intelligence

## Abstract

The growing field of digital disease detection, or epidemic intelligence, attempts to improve timely detection and awareness of infectious disease (ID) events. Early detection remains an important priority; thus, the next frontier for ID surveillance is to improve the recognition and monitoring of drivers (antecedent conditions) of ID emergence for signals that precede disease events. These data could help alert public health officials to indicators of elevated ID risk, thereby triggering targeted active surveillance and interventions. We believe that ID emergence risks can be anticipated through surveillance of their drivers, just as successful warning systems of climate-based, meteorologically sensitive diseases are supported by improved temperature and precipitation data. We present approaches to driver surveillance, gaps in the current literature, and a scientific framework for the creation of a digital warning system. Fulfilling the promise of driver surveillance will require concerted action to expand the collection of appropriate digital driver data.

## Abstract 2

Improved and expanded data collection is required to fulfil the promise of an early-warning digital system.

Unusual infectious disease (ID) events occur when an underlying mix of antecedent epidemiologic drivers provide the necessary conditions for a pathogen to emerge in susceptible populations. These conditions may be driving emergence through a wide variety of mechanisms, such as climate change, industrial development, ecosystem change, and social inequality (*1*). Public health policy has traditionally targeted well-described socioeconomic drivers, such as lack of sanitation, lack of hygiene awareness, and poor access to health care and disease prevention services (e.g., bed nets, vaccinations, and treatments), but researchers have increasingly evaluated the complex interactions among drivers related to globalization, political issues, human susceptibility, and biophysical environmental change (*1*–*8*) (Table).

**Table:** Disease drivers identified in the literature and examples of data availability*

Driver theme (references)	Global data examples†	Regional data examples†
Human susceptibility to infection (*1**,**2**,**4*)	Vaccine rumor surveillance, product distribution data from manufacturers, self-reported immunization status	US influenza vaccination rates, measles vaccination rates from the Mozambique Health Information System
Climate and weather (*1**,**2**,**4*)	Numerous satellite products, National Oceanic and Atmospheric; Administration, Climatic Research Unit, Center for Sustainability and the Global Environment, vulnerability to climate change	Climate data, social media reports of climate and air pollution effects on Twitter and Sina Weibo
Human demographics and behavior (*1**,**2**,**4*)	Night time lights, Gridded population of the world, mobile phone operator data	National census data products, Twitter, world population
Economic development (*1**,**2**,**4*)	International Monetary Fund, World Bank	National departments of economics
Land use and ecosystem changes (*1**,**2**,**4*)	Global agricultural lands, Center for International Earth Science Information Network, Global Forest Change 2000–2012, Global Forest Watch, global livestock distribution densities	National departments of agriculture, croplands in western Africa, Africa mining digital news reports, IMAZON Deforestation Alert System
Technology and industry (*1**,**2**,**4*)	Digital news, United Nations Global Pulse	NA
Human wildlife interaction (*2**,**4*)	Species distribution grids, digital news reports	State-level hunting data
Breakdown of public health measures (*1**,**2**,**4*)	Natural disaster hotspots	News of impending natural disasters (i.e., predicted hurricane landfall)
Poverty and social inequality (*1*)	Center for International Earth Science Information Network, Global Observatory	National census data
War and famine (*1**,**2**,**4*)	Famine early warning system, digital news and social media	Syria Tracker
Lack of political will (*1*)	Historical records, Transparency International, Cline Center for Democracy	NA
International travel and commerce (*1**,**2**,**4*)	Flight and shipping data	Regional distribution data of food products

*The table is purposely not exhaustive but provides a survey of types of available digital data that are associated with different drivers. NA, not applicable. †See Technical Appendix Table for available references.

Whereas early ID research primarily focused on pathogen identification and specific disease ecologies (*9*), researchers are now exploring the multifactorial causes of emergence. No longer is the question “What causes Ebola?” but rather, “Why does an Ebola outbreak occur at a particular time or location?” As a result of this transition in research, global disease event data have grown. The World Health Organization Global Burden of Disease Reports, commissioned in 1992, first demonstrated the feasibility of measuring both global risk and disease occurrence data (*10*). Scientists built on such initial global platforms and began to develop tools to improve reporting and awareness of disease outbreaks among local and global health workers. In the 1990s, digital systems such as ProMED and Global Public Health Intelligence Network demonstrated the utility of real-time digital disease event detection (*11*,*12*). The emergence of these intelligence platforms was followed by a new generation of surveillance tools, such as HealthMap, Biocaster, and MedISys (*13*–*15*).

Behind the development of surveillance tools for ID events, growing research suggests that untapped driver signals could be quantified and monitored to anticipate emergence risk as a new form of epidemic intelligence. Temperature and precipitation data are already used to forecast meteorologically sensitive IDs, but more driver data could support improved predictive models covering a much broader range of IDs. For example, across 397 outbreaks of international concern, as classified by the World Health Organization, nearly 40% were attributed to 1 driver: lack of public health infrastructure (*2*). Further, changes in land use, another known driver, can produce animal–human interfaces ripe for spillover events (*3*,*7*). Indeed, between 1940 and 2005, 60% of emerging ID events were of zoonotic origin and showed a substantial positive correlation with wildlife abundance and diversity (*4*). The logical progression to further strengthen public health infrastructure is to expand surveillance to monitor the full spectrum of ID emergence drivers (Figure 1) (*2*,*16*,*17*).

**Figure 1:**
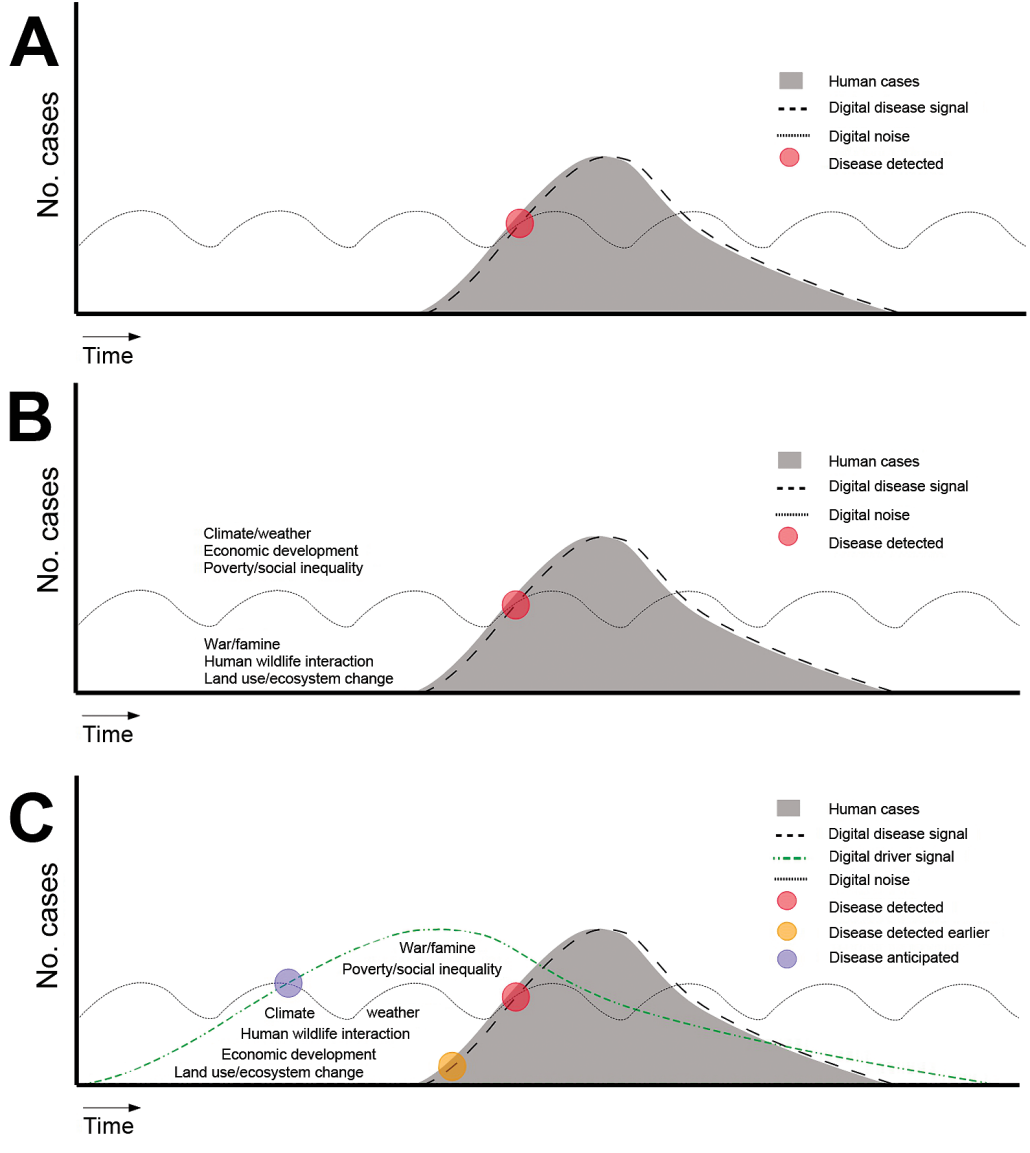
Surveillance and detection of disease by traditional (A, B) and digital (C) detection systems. A) Traditional disease detection, in which a close association exists between the number of cases and the digital disease signal. Disease is detected when the signal exceeds the noise. B) Disease emergence or outbreaks often occur following a driver. Examples of such drivers include climate and weather, economic development, poverty and social inequality, war and famine, human–wildlife interactions, land use and ecosystem changes. C) Detection of disease by using digital techniques. In this system, drivers of disease (not disease) are monitored, essentially to monitor for conditions suitable for disease emergence. Hypothetically, the careful surveillance of drivers that have been separated from digital noise could shorten the time to disease detection (as indicated by the orange dot).

In the historical case of bromeliad malaria (see Drivers in Action and Hindsight section), actions were taken only after astute clinicians recognized the pattern of an ID event, malarial symptoms around cacao farms. Had a driver surveillance system been available, the elevated risk for an outbreak might have been indicated by reports of agricultural land use changes and human movement in conjunction with the underlying poverty and lack of disease prevention practices. Ideally, a warning system would then trigger an active surveillance program, a preemptive investigation to reduce risk factors, or even control measures (Figure 1).

An integrated driver surveillance system has the potential to be an integral tool for analysts and decision makers at 2 stages. First, whereas standard surveillance methods were designed to detect an outbreak, a driver-centric system will provide situational awareness of potentially unhealthy conditions before and following an outbreak. At the pre-outbreak stage, decision makers will have a tool that summarizes risk across data streams for multiple drivers of IDs. Second, at the postoutbreak stage, driver surveillance could result in more efficient resource allocation. Interventions could be tailored to local needs and capacity based on the knowledge of underlying drivers that will vary over space and time (e.g., infrastructure, vaccine coverage, and public health capacity) (*18*).

A warning and response system will require improved understanding of the relationships among specific drivers and ID emergence events. Advancements must also be made in terms of driver data collection. Well-developed datasets regarding climate, land cover change, and population density already exist (e.g., Center for International Earth Science Information Network, Climate Research Unit, and Google Earth Engine), but well-developed datasets are not readily available for drivers associated with human behavior (e.g., open-source curated data on immunization coverage, public health breakdown, sanitation and hygiene, and vector control). Well-developed, readily available datasets are those that provide subcountry level data or higher resolution at regular and frequent intervals with documented and standardized tools that are published and freely available online. Through curation and accessibility, these data and an expanded knowledge of drivers could greatly enhance mathematical models that describe ID transmission and epidemic occurrence. Herein, we survey available digital resources, present a conceptual framework for such a digital disease driver surveillance platform, and discuss opportunities for and obstacles to its successful implementation.

## Drivers in Action and Hindsight

In the 1940s, a malaria epidemic began when economic pressures and poverty led the agriculture sector of Trinidad into the cacao industry, an activity that requires a large human labor force and shade trees. These shade trees supported a bromeliad tank species, a family of plants that contain water-holding structures, creating an ideal breeding site for *Anopheles bellator* mosquitoes and new niches for different mosquitoes within the forest canopy (*19*,*20*). During the epidemic, medical doctors first noted an increase in the prevalence of splenomegaly, an indication of malaria, among schoolchildren correlated with areas cultivating cacao, and that *A. bellator* mosquitoes were only found near the cacao farms. Removal of the bromeliads reduced *A. bellator* mosquito populations and returned malaria rates to prior endemic levels (*20*). In hindsight, the outbreak resulted from the convergence of poverty, commerce, and agricultural and land-use changes; lack of malaria prevention and treatment services; and resulting shifts in local mosquito ecology. Building on our accumulating knowledge of drivers and the digital streams of data that now exist, the public health sector can be provided with early warning tools to recognize when conditions are ripe for disease emergence events, tools that could not have existed in the mid-20th century.

## Identifying Existing Optimal Spatial Data System Components

Driver monitoring requires access to data from multiple domains at sufficient resolution and scale to correlate with known disease events and overcome potential biases. To build a driver surveillance system, issues with data availability and compatibility must be addressed in conjunction with temporal resolution and spatial scale.

**Figure 2:**
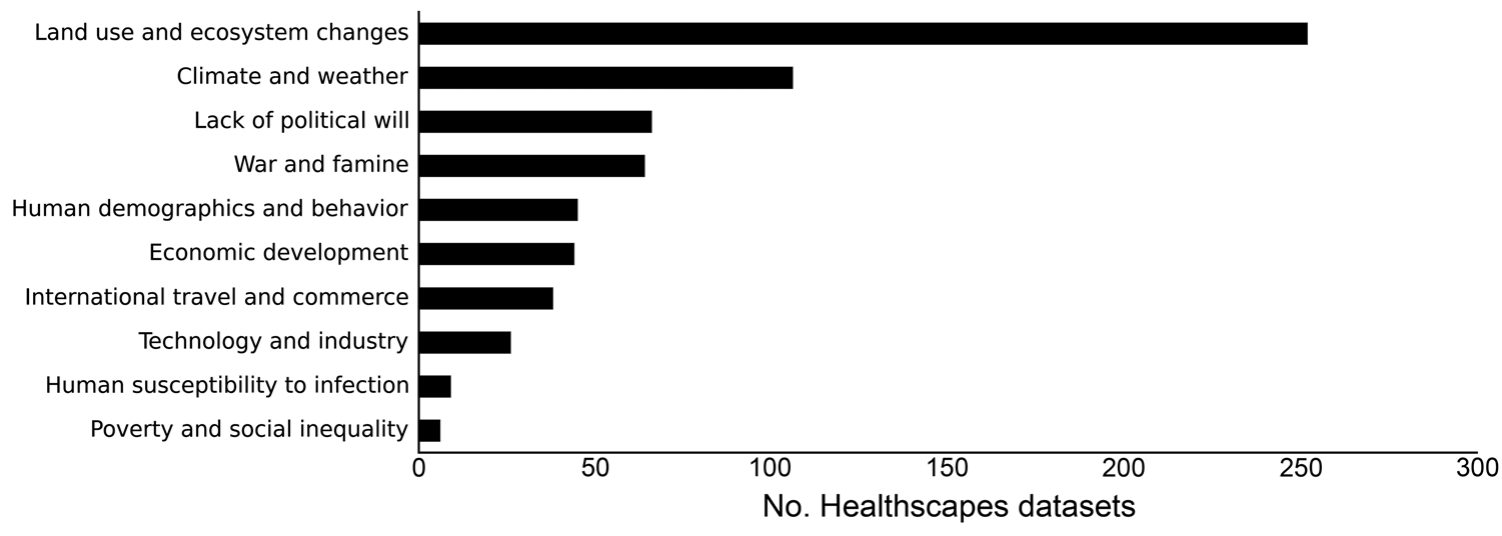
Number of datasets, by disease driver, available globally. The data were collected for the HealthScapes Project (http://healthscapes.io).

Data availability categorized by drivers of interest is shown in Figure 2. The data were collected for the HealthScapes Project (http://healthscapes.io), an initiative launched in 2009 to assemble and collaboratively curate global open data of relevance to the global health research community. Of note, datasets were not readily found for certain drivers (i.e., human wildlife interaction and breakdown of public health measures), and few datasets were found for other drivers (i.e., human susceptibility to infection and poverty and social inequality).

### Availability

The global volume of data is doubling every 2 years (*21*). Although this trend presents an opportunity for analysts, much of the data are either unreduced big data that have become stored at major institutions or transient data that are not stored. Although potentially applicable to geographic, ecologic, and sociologic driver detection, only a fraction of these data have been processed for analysis on current research infrastructure, and even less is publicly accessible. Despite efforts to make data available, few providers have adopted provenance or metadata standards or leveraged mechanisms to facilitate collaboration, such as web services and application programming interfaces.

### Compatibility

Technology trends, software development practices, and disciplinary preferences have resulted in a mosaic of data types and formats. Despite increasing interoperability, propriety obstacles persist, and innovations have isolated valuable legacy data in archaic formats.

### Temporal Resolution

The distribution of data is generally skewed to the present because of increased digitization, the emergence of the Internet, and widespread instrumentation of systems. Research funding levels, political cycles, disruptive events, research trends, individual efforts, and the evolution of organizations all contribute to temporal variability in data resolution.

### Spatial Scale

Few driver datasets of interest have complete global coverage. When considering data storage location as a proxy for spatial coverage, 50% is in North America, 30% in Europe, and 14% in the Asia–Pacific region; the Middle East, Latin America, India, and Africa account for the remaining 6% (*22*). This uneven distribution presents a challenge for monitoring disease in underdescribed regions. Furthermore, when attempting to grid, or rasterize, data at the global scale, researchers may codify incorrect assumptions through interpolation or by applying bias corrections for research effort. The distribution of data is evolving rapidly; if we consider submarine cable traffic rather than storage volume, intra-Asian connections have surpassed trans-Atlantic bandwidth (*23*). It is not yet determined how this shifting flow of information may influence driver coverage and effect digital media surveillance.

## Leveraging Digital Media Reports

Digital media reports already provide a rich source of information on disease outbreaks (*24*) and, likewise, may be useful in monitoring disease drivers to identify periods of increased risk for outbreaks (e.g., news articles about breakdown of public health infrastructure, conflict, and vaccine programs). However, this driver surveillance resource presents a unique mix of opportunities and challenges. On the opportunity side, only a handful of languages account for most content on the Internet, resulting in a distilled set of global languages (*25*). If this pattern continues, only a subset of languages will need surveillance. Furthermore, the ability of natural language processing tools to parse reports for contextual data, such as location information, is improving. On the challenge side, digital media is fleeting and curated datasets are limited. For example, Google News, a popular digital media aggregator, does not provide a list of the sources used in its feed, and, thus, its content could change with time (*26*). Digital media can also miss nondigital disease-related news reports and details due to language or source (e.g., print and audio) limitations (*27*).

An objective evaluation of digital media for driver surveillance will ultimately require an unbiased dataset that links media reports of disease drivers to actual outbreaks that enable determination of the specificity and sensitivity of the system. Notably, it will be difficult to develop such a system because of the circular nature of these data (i.e., the system would be evaluated based on the system used to collect the data). In the absence of independently collected gold standard data, one cannot determine the degree to which unknown unknowns are missed. In summary, more research is needed in evaluating digital media systems and the influence of these potential limitations.

## New Data Technology Solutions for Drivers

Numerous projects have been established as hubs for collecting, cataloging, and sharing global data on a wide variety of potential drivers. Underlying these streams is an increasingly open-data ethic. Numerous repositories of existing data specialize in distributing accessible data formats (e.g., World Organisation for Animal Health’s World Animal Health Information Database, Global Biodiversity Information Facility, Center for International Earth Science Information Network, Google Earth Engine, Gapminder World, European Union BioFresh project, and HealthScapes). Recent Digital Disease Detection conferences have highlighted numerous new data sources, including mobile phone and Internet technology that has enabled real-time syndromic and resource monitoring from social media (Twitter and Facebook), active surveillance (e.g., Flu Near You and Influenzanet), and participatory surveillance (Opendream, SMS for Life, and Saude Na Copa 2014). On the ground and at the source, data loggers and sensors are decreasing in cost and are supported by open-source platforms (e.g., Mataki, Arduino, and Onset HOBOs).

HealthMap is one example of emerging digital surveillance technology that uses a flexible architecture and an automated processing system to target ID events and the wildlife trade (*13*,*28*). This framework could be applied to identify any driver of interest as dictionaries for different languages and subject areas are used; the platform is fundamentally subject- and language-agnostic. This structure enables for inclusion of data not distributed digitally, resulting in the ability to expand overall media coverage. In the near future, developments in optical character and audio language recognition offer the potential for the rapid processing of nondigital media, such as photographs of print media and audio news sources.

Coinciding with the growing number of data sources has been an explosion of visualization, analytical, and hybrid tools, often fueled by the open-source community. Once data are collected, these tools will provide new ways for analysts to convey complex data and ideas, such as driver surveillance trends, to decision makers in a clear and logical format (e.g., Hans Rosling’s Trendalyzer, Technical Appendix Table 2). Cloud-based platforms have evolved from desktop-based Esri software and leverage the open-source framework, social coding, and collaboration to provide researchers new opportunities to combine complex multisource big data with newly created virtual forums (e.g., Google Earth Engine and social modeling platforms). In addition to new analytic techniques, digital driver surveillance can build on advances in the way science is conducted. One example is the Open Science Framework, which has pioneered online project spaces that enable others to make changes or add on to the project without affecting the original (https://osf.io/). Another example is rOpenSci (https://ropensci.org), an effort funded by the Sloan Foundation to wrap scientific application programming interfaces in a manner that is readily accessible to analysts and researchers. This new approach sets the groundwork for collaborative, iterative, and adaptive management of ID driver surveillance models.

## Application of Driver Surveillance for a Novel ID

The emergence of Nipah virus (NiV) on the global scene can be linked to several drivers, all of which could have been monitored and potentially recognized, resulting in quicker containment of outbreaks. The first human NiV cases were reported in 1998 in Malaysia. This initial spillover event has been linked to agricultural intensification where mango trees were planted in close proximity to pigsties (*16*,*29*). This change in practice led to increased contact between pigs and the natural reservoir for NiV, the flying fox, and subsequent introduction of NiV into the pig population (*29*). The continued contact between the bats and pigs resulted in NiV establishment within the pig population and increasing exposure of pig workers to NiV (*29*,*30*).

Perhaps NiV emergence could have been anticipated if a system existed that raised warnings about the coincident land-use change and agricultural intensification. Scientists were eventually able to match the unknown disease affecting humans with NiV and identified risk factors and modes of transmission, but the key connections might have been recognized sooner. Although an isolated change in agricultural practices would not typically warrant an intervention, the emergence of a new porcine respiratory disease in conjunction with the change could have led to closer monitoring. When the initial spillover into the human population occurred, the knowledge of a new porcine disease, changes in driver activity, and the identification of a new virus in bats all occurring within the same region could have individually triggered heightening alerts.

After emergence, driver surveillance would have provided valuable situational insight. In the early stages of the outbreak, there was a mass sell-off of pigs in response to the cluster of cases and the perceived link among farmers between the sick pigs and the cluster (*29*,*31*,*32*). This sell-off resulted in the spread of NiV to southern regions of Malaysia, where there is a dense pig population (*29*). As the outbreak spread southward, the international pig trade moved the virus to Singapore (*32*). Market monitoring could have potentially identified the increase in pig sales, as well as the regional sources and endpoints of traded pigs. If supply chains and the locations that sit at high-risk critical nodes could be identified, increased active surveillance or even preemptive interventions, such as animal culling, could have been deployed.

## Field Deployment as a Two-Way Communication Interface

A successful epidemic surveillance platform should leverage the latest web technologies to support global communication among health workers with near–real-time information on the drivers of disease emergence. It is important that the platform have the capability to capture ground-truthed data from field teams to effectively crowd-source data curation to amplify efforts in the global response to disease emergence. Such open data curation and provisioning services would further help democratize data access to those often most affected by IDs. Core features of the system would include the ability to identify relevant drivers for a disease or region of interest as well as the capacity to monitor spatiotemporal trends for these drivers. A user could use this information to make decisions about allocating funding for health control measures or prioritizing health effect assessments of development initiatives. In the field, a mobile interface to the platform could be used to identify future field sampling sites, intervention options, or changes in drivers. It is essential the platform have the capacity to monitor and overlay multiple drivers, in light of known interactions. The utility of the system would be enhanced by being able to set notifications on drivers of interest that could initiate a response, follow up, or further investigation. A truly dynamic web interface would enable the user to simulate different health outcomes under different scenarios, such as economic policies, control strategies, or climate futures. In anticipation of future ID outbreaks, a well-designed system could be used strategically by decision makers to allocate or predeploy limited human and material resources to risk hotspots. Globally, the system could be used to help orchestrate multinational responses to emerging ID threats, identify priorities, and raise awareness of the role of antecedent drivers in global health.

## Moving toward a Prototype Platform

For a first-generation analysis platform to prove useful, it must provide insight regarding specific drivers and the drivers’ subsequent effect upon current risk. We envision a country-based analysis framework that could comprise 3 components. The first component will measure a nation’s risk for an emerging ID event by establishing driver baselines through the use of historical data or published ratings. The hotspots approach used by Jones et al. (*4*) illustrates the potential of this component, which could be extended from using historical events to using historical driver data to identify geographic locations at risk for ID emergence. Together these baselines would provide a Bayesian prior distribution of risk. The second component will introduce existing and new digital alerts as new evidence for a model. The addition of these alerts would build a risk map according to baseline levels; that is, a driver with a low baseline will not contribute much to changing the overall risk with the addition of a few alerts, but for a driver with a high baseline, the addition of a few driver alerts could push it into a higher risk category. By overlaying the digital disease alerts upon the baseline prior distribution of risk, we can then analyze these data for trends and biases to anticipate future emerging ID events. The third component will involve continually updating beliefs about the distribution of risk that is determined on the basis of new evidence.

Despite our positive outlook on digital driver surveillance, we note several caveats, such as the complexity of causal human–environment interactions (*33*) and the use of models to confront imperfect knowledge. For instance, false-positive signals, when a driver system identifies a strong signal but no disease occurs, are inevitable. Without appropriate user sensitization, this result could lead to misallocation of resources and erosion of trust. Conversely, some disease events might lack any warning signals, as may have been the case with the 2009 pandemic of influenza A(H1N1)pdm09 virus infections. Despite the existence of these problems, we believe they will be alleviated through a better understanding of the disease–driver and driver–driver associations. However, some problems will be unavoidable. For example, a potentially strong limitation would be that infrequent updating of driver data could result in either missing a brief signal or not identifying the signal until it is too late to implement control measures. Ultimately, we must recognize that digital surveillance is one tool among many that can help supplement, but not replace, traditional public health surveillance.

## Conclusions

A digital driver surveillance platform that improves situational awareness by active monitoring of ID events and associated drivers is an obtainable goal for the public health community. We believe that future surveillance platforms will be able to not only describe driver activity in space and time but also indicate driver thresholds, severity, and likely interactions among drivers. Digital media reporting offers tremendous potential to contribute to existing datasets because of its automated ability to scrape news sources for alerts and provide real-time driver data. Channels of communication need to be opened between the data producers, who may not see the wider utility of what is collected, and data consumers, who may not understand barriers with data collection. In addition, funding agencies that support driver-associated data collection efforts need to require that recipients follow best data practices. We recognize the existence of obstacles to developing a digital driver surveillance platform, but as data become more available, compatible, and refined, our ability to overcome systematic biases and sources of error to identify driver activity will become easier. This report is a call to action to improve collection of driver and ID event data to rapidly develop the science and our understanding of relationships between drivers and emerging ID events, and move toward driver-based ID surveillance systems.

Technical Appendix References for data examples in the Table and URLs for web platforms mentioned in the text.
