# Supplementary material for: Drivers of Emerging Infectious Disease Events as a Framework for Digital Detection
Source: Emerg Infect Dis. 2015 Aug;21(8):1285–92. doi: 10.3201/eid2108.141156 (PMC4517741; doi:10.3201/eid2108.141156)
Supplement: Technical Appendix — References for data examples in the Table and URLs for web platforms mentioned in the text. [file 14-1156-Techapp-s1.pdf]

Article DOI: http://dx.doi.org/10.3201/eid2108.141156

Drivers of Emerging Infectious Disease Events as a Framework for Digital Detection

Technical Appendix

Technical Appendix Table 1. References for available data examples found in the Table in the article text*

Driver theme, data example	Citation
Human susceptibility to infection	
Vaccine rumor surveillance	(*1*)
U.S. influenza vaccination rates	(*2*)
Mozambique Health Information System	(*3*)
Climate and weather	
SAGE (Center for Sustainability and Global Environment)	(*4*)
Vulnerability to climate change	(*5*)
Human demographics and behavior	
World Pop	(*6*)
Economic Development	
International Monetary Fund	(*7*)
World Bank	(*8*)
Land use and ecosystem changes	
Global Agricultural Lands	(*9*)
CIESN (Center for International Earth Science Information Network)	(*10*)
Global Forest Change 2000–2012	(*11*)
Global Forest Watch	(*12*)
Croplands in western Africa	(*13*)
IMAZON Deforestation Alert System	(*14*)
Global livestock distributions	(*15*)
Technology and Industry	
United Nations Global Pulse	(*16*)
Human wildlife interaction	
Digital news reports	(*17*)
State-level hunting data	(*18*)
Breakdown of public health measures	
Natural disasters hotspots	(*19*)
Poverty and social inequality	
CIESEN (Center for International Earth Science Information Network)	(*10*)
Global Observatory	(*20*)
War and famine	
Famine early warning system	(*21*)
Syria Tracker	(*22*)
Lack of political will	
Transparency International	(*23*)
Cline Center for Democracy	(*24*)
International travel and commerce	
NA	NA

*NA, none available.

Technical Appendix Table 2. References for web platforms mentioned in the text

Web platform	Web site
FluNearYou	https://flunearyou.org/
Influenzanet	https://www.influenzanet.eu/
OPENDREAM – Thailand	https://www.opendream.co.th/
SMS for Life	http://malaria.novartis.com/innovation/sms-for-life/index.shtml
Saude Na Copa 2014	http://www.saudenacopa.com/dashboard/site/termos
Mataki	http://mataki.org/
Arduino Platform	http://www.arduino.cc/
Onset HOBOs	http://www.onsetcomp.com/
Trendalyzer	http://www.gapminder.org/
Esri	http://www.esri.com/
Google Earth Engine	https://earthengine.google.org/#intro

References

1. HealthMap. Vaccine Sentimeter: Global monitoring of vaccination conversations. 2014 Feb 1 [cited 2014 Jun 25]. http://www.healthmap.org/viss/

2. US Department of Health and Human Services. Interactive mapping tool: live-tracking flu vaccinations of Medicare beneficiaries. 2014 May 23 [cited 2014 Jun 25]. http://www.hhs.gov/nvpo/flu-vaccination-map/

3. World Health Organization. WHO vaccine-preventable diseases: monitoring system. 2014 global summary. 2014 [cited 2014 Jun 25]. http://apps.who.int/immunization_monitoring/globalsummary/estimates?c=MOZ

4. Center for Sustainability and the Global Environment University of Wisconsin–Madison. WHIPS (the Wisconsin Horizontal Interpolation Program for Satellites). 2013 Mar 6 [cited 2014 Jun 25]. http://www.sage.wisc.edu/download/WHIPS/WHIPS.html

5. Wheeler D. Quantifying vulnerability to climate change: implications for adaptation assistance. Washington (DC): Center for Global Development; 2011.

6. GeoData Institute, University of Southampton. WorldPop [cited 2014 Jun 25]. http://www.worldpop.org.uk/

7. International Monetary Fund. Data and statistics. 2014 [cited 2014 Jun 25]. http://www.imf.org/external/data.htm

8. The World Bank. Data. 2014 [cited 2014 Jun 25]. http://data.worldbank.org/

9. LUGE (Land Use and the Global Environment) Lab. Data sets. 2013 May 3 [cited 2014 Jun 25]. http://www.geog.mcgill.ca/~nramankutty/Datasets/Datasets.html

10. NASA's Socioeconomic Data and Applications Center (SEDAC). World Data Center for Human Interactions in the Environment. 2011 [cited 2014 Jun 25]. http://sedac.ciesin.columbia.edu/wdc/datapolicy.jsp

11. Hansen MC, Potapov P, Moore R, Hancher M, Turubanova S, Tyukavina A, et al. High-resolution global maps of 21st-century forest cover change. Science. 2013;342:850–3. PubMed http://dx.doi.org/10.1126/science.1244693

12. World Resource Institute. Global Forest Watch [cited 2014 Jun 25]. http://www.globalforestwatch.org/

13. Ramankutty N. Croplands in West Africa: a geographically explicit dataset for use in models. Earth Interact. 2004;8:1–22. http://dx.doi.org/10.1175/1087-3562(2004)8<1:CIWAAG>2.0.CO;2

14. Institute of Man and Environment in the Amazon. Publications. 2011 [cited 2014 Jun 25]. http://www.imazon.org.br/publicacoes

15. Geo-Wiki. Livestock Geo-Wiki. 2014 [cited 2014 Jun 25]. http://www.livestock.geo-wiki.org/

16. United Nations. United Nations Global Pulse. 2014 [cited 2014 Jun 25]. http://www.unglobalpulse.org/

17. HealthMap. Wildlife Trade. 2014 [cited 2014 Jun 25]. http://www.healthmap.org/wildlifetrade/

18. Illinois Department of Natural Resources. IDNR harvest query. 2014 [cited 2014 Jun 25]. http://www.dnr.illinois.gov/hunting/Pages/IDNRHarvestQuery.aspx

19. Center for Hazards and Risk Research at Columbia University. Core data sets. 2005 [cited 2014 Jun 25]. http://www.ldeo.columbia.edu/chrr/research/hotspots/coredata.html

20. International Peace Institute. Catalogue of indices. 2014 [cited 2014 Jun 25]. http://theglobalobservatory.org/indices.html

21. United States Agency for International Development. Famine Early Warning System Network. [cited 2014 Jun 25]. http://www.fews.net/

22. Syria Tracker [cited 2014 Jun 25]. https://syriatracker.crowdmap.com/

23. Transparency International. 2014 [cited 2014 Jun 25]. http://www.transparency.org/

24. Cline Center for Democracy. Data. 2010 [cited 2014 Jun 25]. http://www.clinecenter.illinois.edu/data/

25. Flunearyou [cited 2015 Mar 3]. https://flunearyou.org/

26. Influenzanet [cited 2015 Mar 3]. https://www.influenzanet.eu/

27. OPENDREAM – Thailand [cited 2015 Mar 3]. https://www.opendream.co.th/

28. SMS for Life [cited 2015 Mar 3]. http://malaria.novartis.com/innovation/sms-for-life/index.shtml

29. Healthy Cup 2014 [cited 2015 Mar 3]. http://www.saudenacopa.com/dashboard/site/termos

30. Mataki [cited 2015 Mar 3]. http://mataki.org/

31. Arduino platform [cited 2015 Mar 3]. http://www.arduino.cc/

32. HOBOs [cited 2015 Mar 3]. http://www.onsetcomp.com/

33. Trendalyzer [cited 2015 Mar 3]. http://www.gapminder.org/

34. ESRI [cited 2015 Mar 3]. http://www.esri.com/

35. Google Earth Engine [cited 2015 Mar 3]. https://earthengine.google.org/#intro
